# The Cardioprotective Effects of Empagliflozin with and Without ACE Inhibition in Chemotherapy-Induced Cardiotoxicity

**DOI:** 10.3390/biomedicines14040903

**Published:** 2026-04-16

**Authors:** Tim Rozovsky, Adrian Siapno, David Y. C. Cheung, Sara M. Telles-Langdon, Allison Ledingham, Paris R. Haasbeek, Lauren Castagna, Lana Mackic, Leah Schwartz, James A. Thliveris, Danielle Desautels, Joerg Herrmann, Davinder S. Jassal

**Affiliations:** 1 Institute of Cardiovascular Sciences, Department of Physiology and Pathophysiology, Max Rady College of Medicine, Rady Faculty of Health Sciences, University of Manitoba, Winnipeg, MB R3E 0T6, Canada; rozovskt@myumanitoba.ca (T.R.); siapnoa1@myumanitoba.ca (A.S.); dcheung@sbrc.ca (D.Y.C.C.); sara.telleslangdo1@ucalgary.ca (S.M.T.-L.); amilosevic@sbrc.ca (A.L.); mackicl@myumanitoba.ca (L.M.); schwartl@myumanitoba.ca (L.S.); 2 Rady Faculty of Health Sciences, University of Manitoba, Winnipeg, MB R3E 3P5, Canada; parishaasbeek@gmail.com (P.R.H.); castagnl@myumanitoba.ca (L.C.); 3 Department of Human Anatomy and Cell Sciences, Max Rady College of Medicine, Rady Faculty of Health Sciences, University of Manitoba, Winnipeg, MB R3E 0J9, Canada; james.thliveris@gmail.com; 4 Section of Hematology and Oncology, Department of Internal Medicine, University of Manitoba, Winnipeg, MB R3A 1R9, Canada; ddesautels@cancercare.mb.ca; 5 Department of Cardiovascular Medicine, Mayo Clinic, Rochester, MN 55905, USA; herrmann.joerg@mayo.edu; 6 Section of Cardiology, Department of Internal Medicine, Max Rady College of Medicine, Rady Faculty of Health Sciences, University of Manitoba, Winnipeg, MB R3A 1R9, Canada; 7 Department of Radiology, Max Rady College of Medicine, Rady Faculty of Health Sciences, University of Manitoba, Winnipeg, MB R3E 0Z2, Canada

**Keywords:** cardiotoxicity, SGLT-2 inhibition, ACE inhibition, cardio-oncology, doxorubicin, trastuzumab

## Abstract

**Background/Objectives:** While doxorubicin (DOX) and trastuzumab (TRZ) improve overall survival in women with breast cancer, these two anti-cancer drugs increase the risk of developing heart failure. As a novel and largely unexplored approach, our aim was to evaluate whether the prophylactic use of the sodium-glucose co-transporter 2 inhibitor empagliflozin (EMPA), with and without the angiotensin converting enzyme inhibitor perindopril (PER), is cardioprotective in preventing DOX + TRZ-mediated cardiotoxicity. **Methods**: In a chronic in vivo murine model, female mice received prophylactic treatment with PER (3 mg/kg), EMPA (10 mg/kg), or EMPA + PER via oral gavage for a total of 3 weeks as a run-in period prior to weekly administration of DOX + TRZ (8 mg/kg and 3 mg/kg, respectively) intraperitoneally for an additional 3 weeks (total of 6 weeks). **Results**: In mice treated with DOX + TRZ, the left ventricular ejection fraction (LVEF) decreased from 75 ± 2% at baseline to 40 ± 4% at week 6. Prophylactic treatment with either PER, EMPA, or EMPA+PER improved LVEF to 58 ± 3%, 66 ± 3%, and 67 ± 4% at week 6, respectively (*p* < 0.05). Histological analyses confirmed significant disruption of myofibrils, vacuolization, and loss of sarcomere integrity in the DOX + TRZ-treated mice. Prophylactic administration with PER, EMPA, or EMPA + PER, however, improved myofibril integrity at week 6 in mice receiving DOX + TRZ. Finally, although the Bax/Bcl-xL ratio was significantly elevated by 1.5-fold in mice treated with DOX + TRZ, this marker of apoptosis was attenuated by prophylactic treatment with either PER, EMPA, or EMPA + PER. **Conclusions**: Prophylactic administration of EMPA mitigated adverse cardiovascular remodeling in a chronic in vivo model of DOX + TRZ-mediated cardiotoxicity.

## 1. Introduction

Cardio-oncology is an emerging discipline that focuses on the prevention, diagnosis, and management of cancer in patients who are at risk of developing cardiovascular complications as a result of their anti-cancer treatment. The most common chemotherapeutic regimen used for treating women with breast cancer includes anthracyclines [1]. Anthracyclines, including doxorubicin (DOX), can cause cardiotoxicity in a dose-dependent manner, affecting up to 10% of the cancer population [2,3]. In women with human epidermal growth factor receptor 2 (HER2)-positive breast cancer, the addition of the monoclonal antibody trastuzumab (TRZ) as a targeted biological therapy improves overall patient morbidity and mortality [4,5,6]. However, the combined use of DOX + TRZ can also increase the risk of cardiotoxicity, affecting up to 25% of women with breast cancer [4,5,6]. A number of mechanisms are involved in DOX + TRZ-mediated cardiotoxicity, including upregulation of inflammation, endoplasmic reticulum (ER) stress, and oxidative stress-induced cell damage and/or death [7,8,9].

Cardiotoxicity remains a serious complication for many anti-cancer therapies, serving as the leading cause of morbidity and mortality in cancer patients [2,3]. Over the past 2 decades, there has been an increased focus on studying guideline-directed medical therapy (GDMT) for heart failure, including renin–angiotensin system (RAS) antagonists and beta blockers, in the prevention of chemotherapy-mediated cardiotoxicity [10,11,12,13]. Despite the encouraging findings of randomized clinical trials (RCTs) including the MANTICORE-101, PRADA, and SAFE trials in the breast cancer population, refs. [10,11,12,13] show that consensus guidelines do not routinely recommend these agents in the prevention of cancer therapy-related cardiac dysfunction (CTRCD) [14]. In lieu of RAS antagonists and beta-blockers, which are associated with several undesirable side effects including hypotension, cough, hyperkalemia, fatigue, and/or depression, recent retrospective studies have suggested that sodium-glucose co-transporter 2 inhibitors (SGLT2i) may be effective in the prevention of CTRCD [15,16].

SGLT2i are a class of oral anti-hyperglycemic agents, originally developed and approved for the treatment of type 2 diabetes mellitus (T2DM) [17]. Several RCTs have demonstrated that SGLT2i can also reduce the risk of heart failure associated hospitalization and mortality in patients with and without diabetes [18,19,20]. Although SGLT2i are now recommended as first-line agents in the GDMT for heart failure, ref. [21] indicates that recent basic science studies have focused on these agents in the prevention of CTRCD [22,23,24,25]. To date, four pre-clinical studies have confirmed the cardioprotective role of SGLT2i in the prevention of DOX-mediated cardiotoxicity in a murine model [22,23,24,25]. As DOX is frequently used with TRZ in women with HER2 positive breast cancer, further studies are required to evaluate the cardioprotective role of SGLT2i in this setting. The SGLT2i empagliflozin (EMPA) was selected for this study since it has robust clinical evidence regarding its efficacy and safety in the heart failure setting, with the highest affinity for binding SGLT2 [26].

The aim of our study was to evaluate whether the prophylactic use of the SGLT2i EMPA, with and without the angiotensin converting enzyme inhibitor perindopril (PER), is cardioprotective in preventing DOX + TRZ-mediated cardiotoxicity in a chronic in vivo female murine model.

## 2. Materials and Methods

### 2.1. Experimental Animal Model

**Figure 1:**
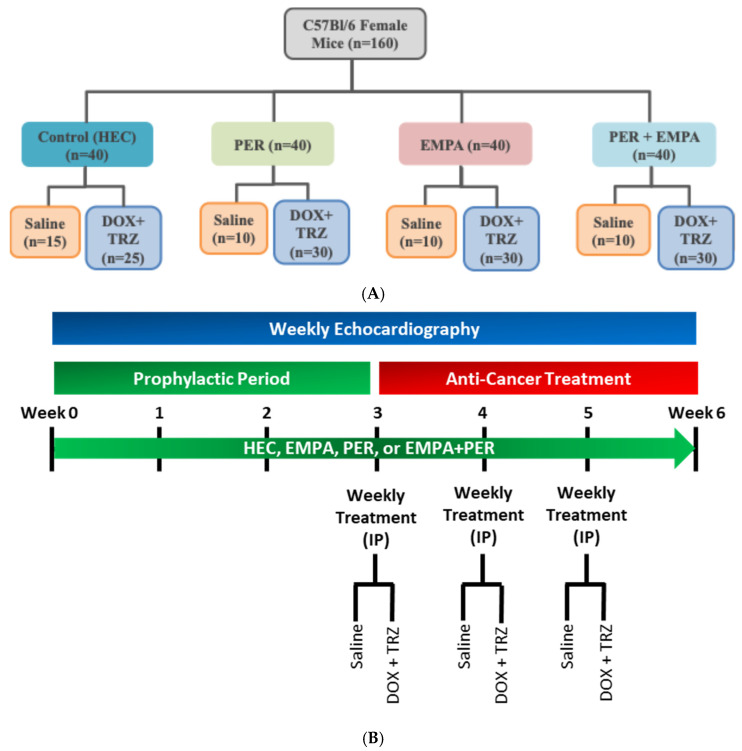
(**A**) Experimental randomization. A total of 160 WT C57Bl/6 female mice (12–15 weeks old; Jackson Laboratories, ME, US) were randomized to the various groups. Mice received prophylactic treatment with 0.5% HEC (n = 40), PER (3 mg/kg/day) (n = 40), EMPA (10 mg/kg/day) (n = 40), or EMPA + PER (n = 40) for a total of 3 weeks as a run-in period prior to administration of 0.9% saline (n = 40) or DOX (8 mg/kg) + TRZ (3 mg/kg) (n = 120) and continued their initial treatment for an additional 3 weeks (total of 6 weeks). At the end of weeks 3, 4, and 5, mice received weekly treatment with DOX + TRZ via i.p. injection to create a chronic in vivo murine model of chemotherapy-induced cardiotoxicity. (**B**) Experimental timeline. Mice were randomized to (i) 0.5% HEC (n = 40); (ii) PER (3 mg/kg/day) (n = 40); (iii) EMPA (10 mg/kg/day) (n = 40); or (iv) EMPA + PER (n = 40) prophylactic treatment groups, receiving the respective treatment via oral gavage daily for the entire 6 weeks of the study. After the prophylactic period (3 weeks), mice received 0.9% saline (n = 40) or DOX (8 mg/kg) + TRZ (3 mg/kg) as an anti-cancer treatment for the next 3 weeks. Cardiac function was assessed weekly using non-invasive TTE.

A total of 160 wild-type C57Bl/6 female mice (12–15 weeks old; Jackson Laboratories, Bar Harbor, ME, USA) were used for this study, randomized into pre-specified groups (Figure 1A). Mice had *ad libitum* access to water and a regular chow diet and were maintained on a 12 h day/night cycle. Mice received prophylactic treatment with a vehicle (VEH) control [(0.5% hydroxyethyl-cellulose (HEC)] [26], EMPA (10 mg/kg/day) [22,25,27], PER (3 mg/kg/day) [28,29], or EMPA + PER via oral gavage for a total of 3 weeks as a run-in period prior to administration of saline or DOX + TRZ for a total of 6 weeks. At the end of weeks 3, 4, and 5, mice received weekly treatment with DOX (8 mg/kg) + TRZ (3 mg/kg) intraperitoneally (i.p.) to create a chronic in vivo murine model of chemotherapy-induced cardiotoxicity, as previously validated by our group and others (Figure 1B) [28,29,30]. The various drug doses of EMPA, PER, DOX, and TRZ listed above, with maximal pharmacological effects, have been validated by our research group and others [22,25,27,28,29].

### 2.2. Murine Echocardiography

Serial non-invasive transthoracic echocardiography (TTE) was performed weekly on awake mice to evaluate cardiovascular remodeling, as previously described [22,28,29,30]. Images acquired in the parasternal long axis view were analyzed to calculate the left ventricular ejection fraction (LVEF). Images acquired in the parasternal short axis (PSAX) M-mode view were analyzed to calculate heart rate, interventricular septal wall thickness (IVS), posterior wall thickness (PWT), and left ventricular end-diastolic diameter (LVEDD), as previously described [22,28,29,30]. The EchoPAC PC software (Vivid 7, version 112, GE Medical Systems) was used for offline analysis of all echocardiographic studies. Echocardiographic data collection and analyses were conducted by 2 observers (TR and DJ) blinded to the various treatment groups.

A total of 30 mice were randomly chosen from the various treatment groups to assess the variability of LVEF. Intra-observer variability was evaluated by a single observer (DJ) who performed independent measurements of LVEF on 2 separate days, 2 weeks apart. Inter-observer variability was determined by 2 independent observers (TR and DJ). Intra- and inter-observer variations were defined as the difference between the 2 observations divided by the mean of the observations and expressed as absolute numbers.

### 2.3. Histological Analysis

Cardiac tissues were prepared for electron microscopy (EM) in a subset of mice from each group (n = 6). A total of 3 blocks were selected for EM at random from the left ventricle of each animal. LV tissue was prepared in accordance with lab-established protocols and utilized for histological examination [29]. Tissue for EM was sectioned, cut into 0.5 mm^2^ pieces, fixed in a 1:1 ratio of 0.2 M Sorensen’s buffer and 6% glutaraldehyde for 3 h, rinsed and stored overnight at 4 °C in a 5% sucrose in 0.1 M Sorensen’s buffer, followed by post-fixation with 1% osmium tetroxide in 0.1 M Sorensen’s buffer for 2 h at room temperature. Tissues were then dehydrated in increasing ethanol concentrations and embedded in Epon 812. Finally, tissue sections were stained with uranyl acetate and lead citrate. To eliminate observer bias, grids were coded without prior knowledge of their origin. The degree of cellular integrity was then determined using digital pictures captured with a Philips CM12 electron microscope [29].

### 2.4. Western Blot Analyses

Western blot analyses were performed in a subset of mice from each group. Western blot analyses was performed to quantify Bcl-2 associated X protein (Bax: a pro-apoptotic biomarker), B-cell lymphoma extra-large (Bcl-xL: an anti-apoptotic biomarker), Bcl-2 interacting protein 3 (Bnip-3; a marker of mitochondrial regulation), 78 kDa glucose-regulated protein (GRP78; a marker of ER stress), and protein disulfide isomerase (PDI; a marker of ER stress). LV tissue samples were flash frozen in liquid nitrogen before being crushed and homogenized in a radioimmunoprecipitation assay lysis buffer containing phosphatase and protease inhibitors to isolate total cellular protein. Samples were then centrifuged to remove cellular debris before the supernatants were collected as protein lysates. A Bradford protein assay was then used to quantify protein concentrations. All protein loading samples were prepared to contain 30 μg of protein in a 1x Laemmli buffer containing 2-mercaptoethanol. Protein detection was performed in a non-blinded manner by applying an enhanced chemiluminescence substrate to the blots, where images were then captured using a BioRad ChemiDoc Imaging System. Protein band intensity was then measured using Densitometric analysis.

### 2.5. Statistical Analysis

The statistical analyses were performed using the software packages IBM SPSS 24.0 and GraphPad Prism 5. Data is presented as mean ± standard deviation (SD) unless otherwise specified. Echocardiographic analyses were carried out using analysis of variance (ANOVA) and Dunnet’s post hoc test. Mann–Whitney and Kruskal–Wallis tests were used for non-parametric score comparisons between groups during histological examination. The values ranged from 0 to 5, with 0 denoting no tissue injury and 5 indicating severe damage. Western blot analytical data was reported as mean ± SD. For post hoc analysis, repeated measures of one-way ANOVA were employed to determine the significance of independent variables. Results with *p* < 0.05 were considered significant.

## 3. Results

### 3.1. Murine Echocardiography

**Figure 2:**
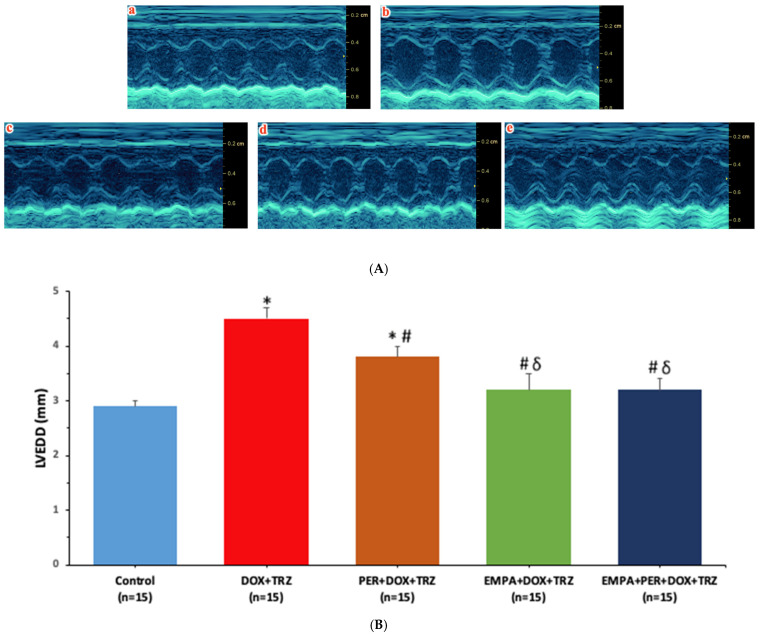
(**A**) Representative echocardiography images acquired in PSAX M-mode view. (**a**) Control; (**b**) DOX + TRZ; (**c**) PER + DOX + TRZ; (**d**) EMPA + DOX + TRZ; and (**e**) EMPA + PER + DOX + TRZ. (**B**) Changes in LVEDD in mice prophylactically administered with PER, EMPA, or EMPA + PER prior to treatment with DOX + TRZ. * *p* < 0.05 DOX + TRZ or PER + DOX + TRZ vs. control. ^#^ *p* < 0.05 PER + DOX + TRZ or EMPA + DOX + TRZ or EMPA + PER + DOX + TRZ vs. DOX + TRZ. ^δ^ *p* < 0.05 EMPA + DOX + TRZ or EMPA + PER + DOX + TRZ vs. PER + DOX + TRZ.

At baseline, the heart rate and LV cavity dimensions were comparable in each study group (Table 1). In mice treated with DOX + TRZ, the LVEDD increased from 2.8 ± 0.1 mm at baseline to 4.5 ± 0.2 mm at week 6 (*p* < 0.05). In mice prophylactically treated with PER, EMPA, or EMPA + PER, the LVEDD values were 3.8 ± 0.2 mm, 3.2 ± 0.3 mm, and 3.2 ± 0.2 mm, respectively, at week 6 (Table 1). In mice treated with DOX + TRZ, prophylactic treatment with EMPA or EMPA + PER was superior to PER alone in mitigating adverse LV remodeling (Figure 2).

**Figure 3:**
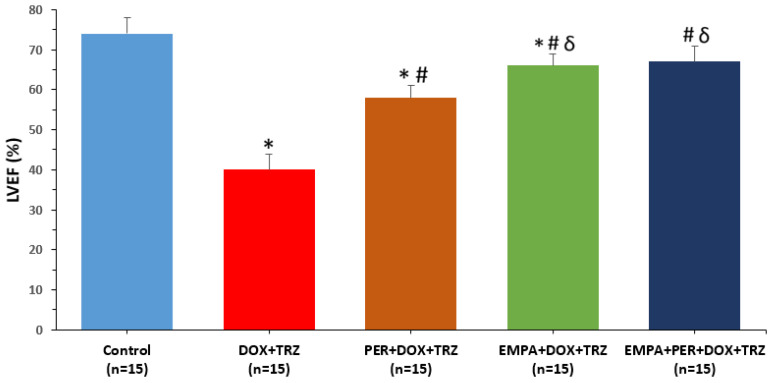
Changes in LVEF in mice prophylactically administered with PER, EMPA, or EMPA + PER prior to treatment with DOX + TRZ. * *p* < 0.05 DOX + TRZ or PER + DOX + TRZ or EMPA + DOX + TRZ vs. control. ^#^ *p* < 0.05 PER + DOX + TRZ or EMPA + DOX + TRZ or EMPA + PER + DOX + TRZ vs. DOX + TRZ. ^δ^ *p* < 0.05 EMPA + DOX + TRZ or EMPA + PER + DOX + TRZ vs. PER + DOX + TRZ.

In mice treated with DOX+TRZ, the LVEF decreased from 75 ± 2% at baseline to 40 ± 4% at week 6. Prophylactic treatment with either PER, EMPA, or EMPA+PER was cardioprotective with LVEF values of 58 ± 3%, 66 ± 3%, and 67 ± 4%, respectively (*p* < 0.05) (Table 1). Prophylactic treatment with EMPA or EMPA + PER was superior to PER alone in preventing LV systolic dysfunction in mice treated with DOX + TRZ (Figure 3). There was minimal intra-observer and inter-observer variability for the LVEF measurements (0.6 ± 0.1 and 1.2 ± 0.2, respectively).

### 3.2. Histology

**Figure 4:**
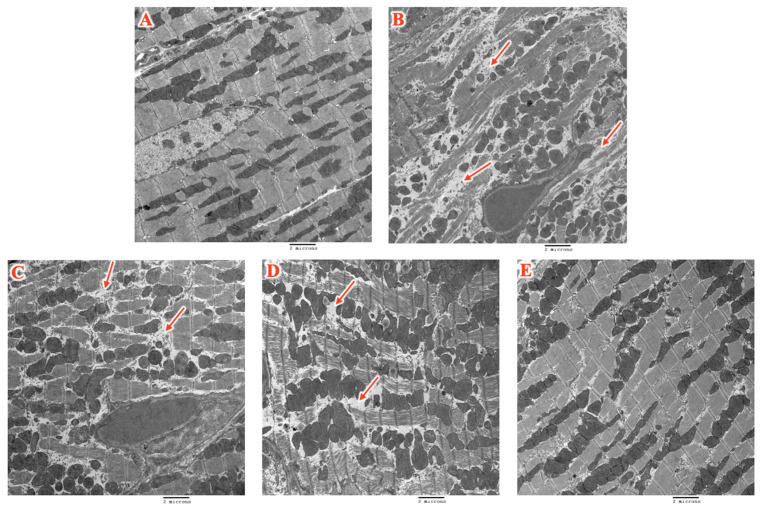
Electron microscopy slides representative of the cell morphology changes for each treatment group. Arrows mark regions showing myofibrillar degradation, vacuolization, and disrupted sarcomere structure. The number of arrows reflects the relative severity of damage. (**A**) Control; (**B**) DOX + TRZ; (**C**) PER + DOX + TRZ; (**D**) EMPA + DOX + TRZ; and (**E**) EMPA + PER + DOX + TRZ.

As compared to the control, EM analyses confirmed significant disruption of myofibrils, vacuolization, and loss of sarcomere integrity in the DOX + TRZ-treated mice (Figure 4A,B). Prophylactic treatment with PER, EMPA, or EMPA + PER improved myofibril integrity at week 6 in mice receiving DOX + TRZ (Figure 4C–E). The prophylactic combination of EMPA + PER showed the greatest benefit in preventing adverse cardiovascular remodeling (Figure 4E).

### 3.3. Western Blot Analyses

**Figure 5:**
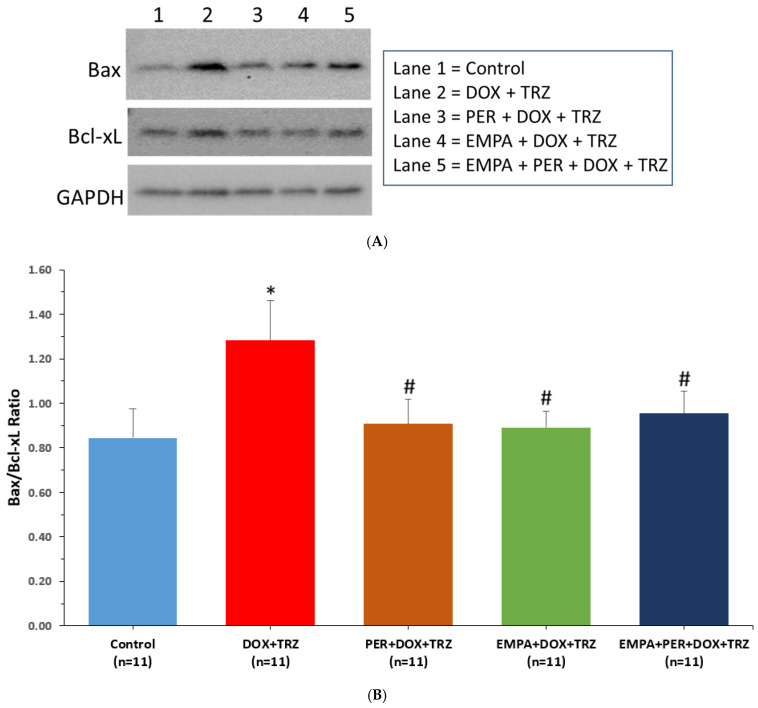
Western blot Bax/Bcl-xL expression. (**A**) Representative Western blot. (**B**) Changes in Bax/Bcl-xL expression in mice from our 5 treatment groups at the end of the study. * *p* < 0.05 DOX + TRZ vs. control. # *p* < 0.05 PER + DOX + TRZ or EMPA + DOX + TRZ or EMPA + PER + DOX + TRZ vs. DOX + TRZ.

In mice treated with DOX+TRZ, there was a 1.5-fold increase in Bax/Bcl-xL expression as compared to healthy control mice (*p* < 0.05) (Figure 5). Elevations in the oxidative stress-induced apoptosis biomarker ratio were significantly downregulated in mice prophylactically treated with PER, EMPA, or EMPA + PER (*p* < 0.05). There were no significant differences in Caspase-3, Bnip-3, GRP78, and PDI expression between all five study groups.

## 4. Discussion

The prevention of CTRCD is a primary focus in the discipline of cardio-oncology. In our chronic in vivo murine model of DOX + TRZ-induced cardiotoxicity, prophylactic administration of EMPA or EMPA + PER was superior to PER alone in preventing adverse cardiovascular remodeling. Our study demonstrated that the prophylactic administration of EMPA and EMPA + PER: (i) mitigated adverse LV cavity remodeling; (ii) decreased cardiomyocyte disruption of myofibrils, vacuolization, and loss of sarcomere integrity; and (iii) attenuated oxidative stress and apoptosis in a chronic in vivo female murine model of DOX + TRZ-mediated cardiotoxicity.

In the field of cardio-oncology, the administration of DOX leads to detrimental cardiovascular remodeling, which may be prevented by SGLT2i through molecular pathways that were previously shown to reduce perivascular and interstitial fibrosis, attenuate ER stress, and lower inflammatory markers [22,23,24,25]. In a 5-week study by Sabatino et al., the prophylactic administration of EMPA improved LVEF from 49% in DOX-treated mice to 61% [22]. Although the 6-week study by Oh et al. did not demonstrate any significant improvement in LVEDD or LVEF values following treatment with EMPA in DOX-treated mice, there was a decrease in LV end-systolic diameter, indicating a degree of cardioprotection [23]. Chang et al. pre-treated diabetic rats with dapagliflozin for 6 weeks followed by 4 weeks of DOX treatment. In their study, the rats demonstrated a small (approximately 9%) but significant improvement in LVEF following the prophylactic treatment with the SGLT2i dapagliflozin [24]. Finally, Quagliariello et al. pre-treated non-diabetic mice with EMPA for 3 days followed by 7 days of DOX + EMPA. In their acute model of DOX-mediated cardiotoxicity, EMPA preserved LVEF (88%) as compared to DOX-only-treated mice (81%) [25]. Collectively, these four murine studies demonstrate the cardioprotective role of SGLT2i in preventing adverse cardiovascular remodeling in DOX-mediated cardiotoxicity [22,23,24,25].

As a complement to these pre-clinical studies that evaluated the prophylactic role of SGLT2i in the prevention of DOX-mediated cardiotoxicity, our study extends these findings to a more clinically relevant DOX + TRZ model. Furthermore, we uniquely included an ACE inhibitor, PER, as a standard-of-care comparator, whereas prior studies used either vehicle [23,24,25] or a non-cardioprotective agent like furosemide [22]. In our chronic in vivo study, prophylactic administration of EMPA, PER, and EMPA + PER prevented adverse LV remodeling. While prophylactic treatment with PER alone improved LVEDD by approximately 16%, the EMPA and EMPA + PER groups demonstrated a greater improvement of approximately 29%. (Figure 3). Prophylactic treatment with PER improved LVEF by 45%, EMPA improved LVEF by 65%, and the combination of EMPA + PER improved LVEF by 68% (Figure 4). Our study is novel in demonstrating that although the ACEi PER prevented adverse cardiovascular remodeling in mice receiving DOX + TRZ, EMPA and EMPA + PER were more cardioprotective. These findings confirm the cardioprotective benefits of SGLT2i observed in prior DOX-only models [22,23,24,25], with and without concurrent ACE inhibition. Although these results demonstrate potent cardioprotection, the absolute magnitude of effect should be interpreted with caution when extrapolating to the multi-factorial, slower-progressing clinical cardiotoxicity setting.

In addition to the echocardiographic changes observed in DOX + TRZ-mediated cardiotoxicity, both anti-cancer agents are associated with characteristic histopathological changes in cardiac tissue. The co-administration of DOX + TRZ results in loss of cardiomyocyte integrity, a reduced number of functional mitochondria, mitochondrial swelling, and cytoplasmic vacuolization [29,31,32]. Notably, previous murine studies in DOX-treated mice demonstrated that these pathological changes may be prevented with the prophylactic administration of SGLT2i [22]. In a in vivo murine study by Sabatino et al., histological analysis using hematoxylin and eosin staining as well as Masson’s trichrome staining revealed that DOX treatment induced significant myocardial fibrosis, myofibrillar disarray, and vacuolization. However, mice pre-treated with EMPA exhibited a 50% reduction in myocardial fibrosis as compared to the untreated group, along with improved preservation of myocardial structure. EMPA also mitigated the loss of cardiac fibers and reduced myocardial disarray [22]. In our study, we confirmed that mice treated with DOX + TRZ demonstrated significant myofibril disruption, vacuolization, and loss of sarcomere integrity, similar to previous studies (Figure 5) [22,29,31,32]. As a novel finding, our study revealed that PER and EMPA attenuated DOX + TRZ-induced myocyte damage, with the combination of EMPA + PER demonstrating the greatest benefit in preserving overall myocardial architecture. This finding suggests an additive effect of EMPA and PER in preventing histopathological alterations in cardiac tissue exposed to DOX + TRZ treatment.

The pathogenesis of DOX + TRZ-mediated cardiotoxicity is complex and multifaceted. It involves activation of inflammatory pathways, induction of ER stress, and elevated production of reactive oxygen species, leading to apoptosis. This pattern of cell death is primarily regulated by the family of Bcl-2 proteins [8]. Anthracyclines lead to the production of reactive oxygen species, activating the mitogen-activated protein kinase (MAPK) pathway, leading to upregulation of the pro-apoptotic Bax protein and downregulation of the anti-apoptotic Bcl-xL protein [7,33]. An increased Bax/Bcl-xL ratio promotes mitochondrial outer membrane permeabilization, leading to the escape of cytochrome c into the cytoplasm, which activates caspases that drive the apoptotic cascade [7,33]. Pre-clinical investigations demonstrate that SGLT2i can attenuate DOX-induced cardiotoxicity by modulating apoptosis [24,25]. Chang et al. demonstrated that DOX treatment approximately doubled the expression of both the pro-apoptotic Bax and cleaved caspase 3 proteins in diabetic rats [24]. Prophylactic treatment with the SGLT2i dapagliflozin, however, decreased these protein markers to levels comparable with control animals [24]. The anti-apoptotic Bcl-2 protein was suppressed by DOX treatment, but its expression in the SGLT2i pre-treated group was found to be similar to that of control rats [24]. Similarly, Quagliariello et al. demonstrated an increase in caspase 3 expression in cardiac tissues from DOX-treated non-diabetic mice. Pre-treatment with EMPA reduced this apoptotic marker by approximately 48% [25].

In our in vivo model of DOX + TRZ-mediated cardiotoxicity, proteomic markers of oxidative stress were partially mitigated by pre-treatment with the combination of EMPA + PER. Our study revealed that the Bax/Bcl-xL ratio, a key indicator of apoptosis induced by oxidative stress, was markedly increased in mice subjected to DOX + TRZ treatment, corroborating previous findings in a DOX-only model [24]. Specifically, these mice exhibited a 1.5-fold increase in Bax/Bcl-xL expression ratio as compared to controls (Figure 5B), signaling a heightened susceptibility to apoptosis within this group. Conversely, this rise in the pro-apoptotic Bax/Bcl-xL ratio was substantially mitigated in animals receiving prophylactic administration of PER, EMPA, or EMPA + PER. This data suggests that DOX + TRZ likely inflict damage upon mitochondria, disrupting the equilibrium of Bcl-2 family proteins in favor of apoptosis. The cardioprotective effects observed with PER, EMPA, and their combined use may stem from their capacity to counteract oxidative stress or directly shield mitochondria, thereby tempering this apoptotic signaling and enhancing cardiomyocyte survival. In fact, our study is the first to demonstrate the cardioprotective effects of an ACEi on preventing increases in the Bax/Bcl-xL ratio in a model of chemotherapy-induced cardiotoxicity, which were similar to SGLT2 inhibition. Despite these findings, other proteomic markers which include Capase-3, Bnip-3, GRP78, and PDI were not significantly altered with DOX + TRZ treatment in our study, which limits the mechanistic breadth of our study. This highlights the need for further investigation into the molecular pathways leading to cardiomyocyte dysfunction in DOX + TRZ-treated mice.

There are limitations to our study. First, our model used only female mice, and while breast cancer predominantly affects women, it also occurs in men. As such, the potential cardioprotective effects of EMPA should also be evaluated in a male murine model [34]. Another limitation is that DOX and TRZ were administered concurrently in our study to enhance the cardiotoxic side effects of these two anti-cancer agents in a murine model. In the clinical setting, these anti-cancer drugs are administered sequentially in women with breast cancer [35,36]. In order to reproduce a model where DOX and TRZ are administered sequentially, a future basic science study would need to be extended to at least 12 weeks of duration. Finally, our murine model involved healthy, cancer-free mice that received DOX + TRZ to induce cardiotoxicity. While we demonstrated the cardioprotective effects of EMPA, we did not assess whether the SGLT2i affects the anti-tumor effects of DOX + TRZ. Before EMPA is incorporated as standard pharmacological therapy in the prevention of CTRCD in cancer patients, it is important to ensure that the SGLT2i does not affect the anti-tumor properties of DOX + TRZ.

## 5. Conclusions

The prophylactic administration of the SGLT2i EMPA mitigated adverse cardiovascular remodeling in a chronic in vivo model of DOX+TRZ-mediated cardiotoxicity. These basic science findings highlight the potential role of SGLT2 inhibition as a cardioprotective preventative strategy in the clinical setting of CTRCD, warranting future clinical RCTs.

## Figures and Tables

**Table 1:** Echocardiographic parameters of C57Bl/6 mice receiving prophylactic treatment with EMPA, PER, or EMPA + PER followed by saline or DOX + TRZ. Baseline and week 6 measures with *p*-values. DOX, doxorubicin; EMPA, empagliflozin; HR, heart rate; IVS, Interventricular septum; LVEDD, left ventricular end-diastolic diameter; LVEF, left ventricular ejection fraction; PER, perindopril; PWT, posterior wall thickness; TRZ, trastuzumab. The values are presented as mean ± SD. * *p* < 0.05 comparing DOX + TRZ, PER + DOX + TRZ, and EMPA + DOX + TRZ vs. control at week 6. ^#^ *p* < 0.05 comparing PER + DOX + TRZ, EMPA + DOX + TRZ, and EMPA + PER + DOX + TRZ vs. DOX + TRZ at week 6. ^δ^ *p* < 0.05 comparing EMPA + DOX + TRZ and EMPA + PER + DOX + TRZ vs. PER + DOX + TRZ at week 6.

Parameter	Group	Baseline	Week 6	*p*-Value
HR (bpm)	Control (n = 15)	697 ± 5	692 ± 7	0.91
	DOX + TRZ (n = 15)	691 ± 4	690 ± 5	0.88
	PER + DOX + TRZ (n = 15)	688 ± 5	682 ± 7	0.84
	EMPA + DOX + TRZ (n = 15)	690 ± 7	694 ± 6	0.82
	EMPA + PER + DOX + TRZ (n = 15)	682 ± 4	688 ± 4	0.86
IVS (mm)	Control (n = 15)	0.81 ± 0.02	0.81 ± 0.01	0.98
	DOX + TRZ (n = 15)	0.81 ± 0.02	0.81 ± 0.01	0.98
	PER + DOX + TRZ (n = 15)	0.82 ± 0.01	0.82 ± 0.02	0.96
	EMPA + DOX + TRZ (n = 15)	0.81 ± 0.02	0.81 ± 0.01	0.98
	EMPA + PER + DOX + TRZ (n = 15)	0.82 ± 0.01	0.82 ± 0.02	0.97
PWT (mm)	Control (n = 15)	0.81 ± 0.01	0.82 ± 0.01	0.97
	DOX + TRZ (n = 15)	0.82 ± 0.02	0.81 ± 0.01	0.96
	PER + DOX + TRZ (n = 15)	0.81 ± 0.01	0.82 ± 0.02	0.96
	EMPA + DOX + TRZ (n = 15)	0.82 ± 0.02	0.81 ± 0.01	0.97
	EMPA + PER + DOX + TRZ (n = 15)	0.82 ± 0.01	0.82 ± 0.02	0.97
LVEDD (mm)	Control (n = 15)	2.8 ± 0.1	2.9 ± 0.1	0.78
	DOX + TRZ (n = 15)	2.8 ± 0.1	4.5 ± 0.2 *	<0.05
	PER + DOX + TRZ (n = 15)	2.8 ± 0.2	3.8 ± 0.2 *^#^	<0.05
	EMPA + DOX + TRZ (n = 15)	2.8 ± 0.1	3.2 ± 0.3 ^#δ^	<0.05
	EMPA + PER + DOX + TRZ (n = 15)	2.8 ± 0.2	3.2 ± 0.2 ^#δ^	<0.05
LVEF (%)	Control (n = 15)	73 ± 4	74 ± 4	0.94
	DOX + TRZ (n = 15)	75 ± 2	40 ± 4 *	<0.05
	PER + DOX + TRZ (n = 15)	74 ± 4	58 ± 3 *^#^	<0.05
	EMPA + DOX + TRZ (n = 15)	74 ± 3	66 ± 3 *^#δ^	<0.05
	EMPA + PER + DOX + TRZ (n = 15)	74 ± 3	67 ± 4 ^#δ^	<0.05

## Data Availability

The original contributions presented in this study are included in the article. Further inquiries can be directed to the corresponding author.

## Abbreviations

The following abbreviations are used in this manuscript:

DOX	Doxorubicin
TRZ	Trastuzumab
EMPA	Empagliflozin
PER	Perindopril
LVEF	Left ventricular ejection fraction
HER2	Human epidermal growth factor receptor 2
ER	Endoplasmic reticulum
GDMT	Guideline-directed medical therapy
RAS	Renin–angiotensin system
RCTs	Randomized clinical trials
CTRCD	Cancer therapy-related cardiac dysfunction
SGLT2i	Sodium-glucose co-transporter 2 inhibitors
T2DM	Type 2 diabetes mellitus
VEH	Vehicle
HEC	Hydroxyethyl-cellulose
TTE	Transthoracic echocardiography
PSAX	Parasternal short axis
IVS	Interventricular septal wall thickness
PWT	Posterior wall thickness
LVEDD	Left ventricular end-diastolic diameter
EM	Electron microscopy
LV	Left ventricular
Bax	Bcl-2 associated X protein
Bcl-xL	B-cell lymphoma extra-large
Bnip-3	Bcl-2 interacting protein 3
GRP78	78 kDa glucose-regulated protein
PDI	Protein disulfide isomerase
RT	Room temperature
CST	Cell Signaling Technology
GAPDH	Glyceraldehyde 3-phosphate dehydrogenase
SD	Standard deviation
ANOVA	Analysis of variance
MAPK	Mitogen-activated protein kinase
